# Supplementary material for: The interactome of intact mitochondria by cross-linking mass spectrometry provides evidence for coexisting respiratory supercomplexes
Source: Mol Cell Proteomics. 2017 Dec 8;17(2):216–32. doi: 10.1074/mcp.RA117.000470 (PMC5795388; doi:10.1074/mcp.RA117.000470)
Supplement: Supplemental Data [file supp_RA117.000470_133922_0_supp_23978_fzffwf.zip › spectra_annotation/mito_spectra_annotation/356-1-3-1-3-1.pdf]

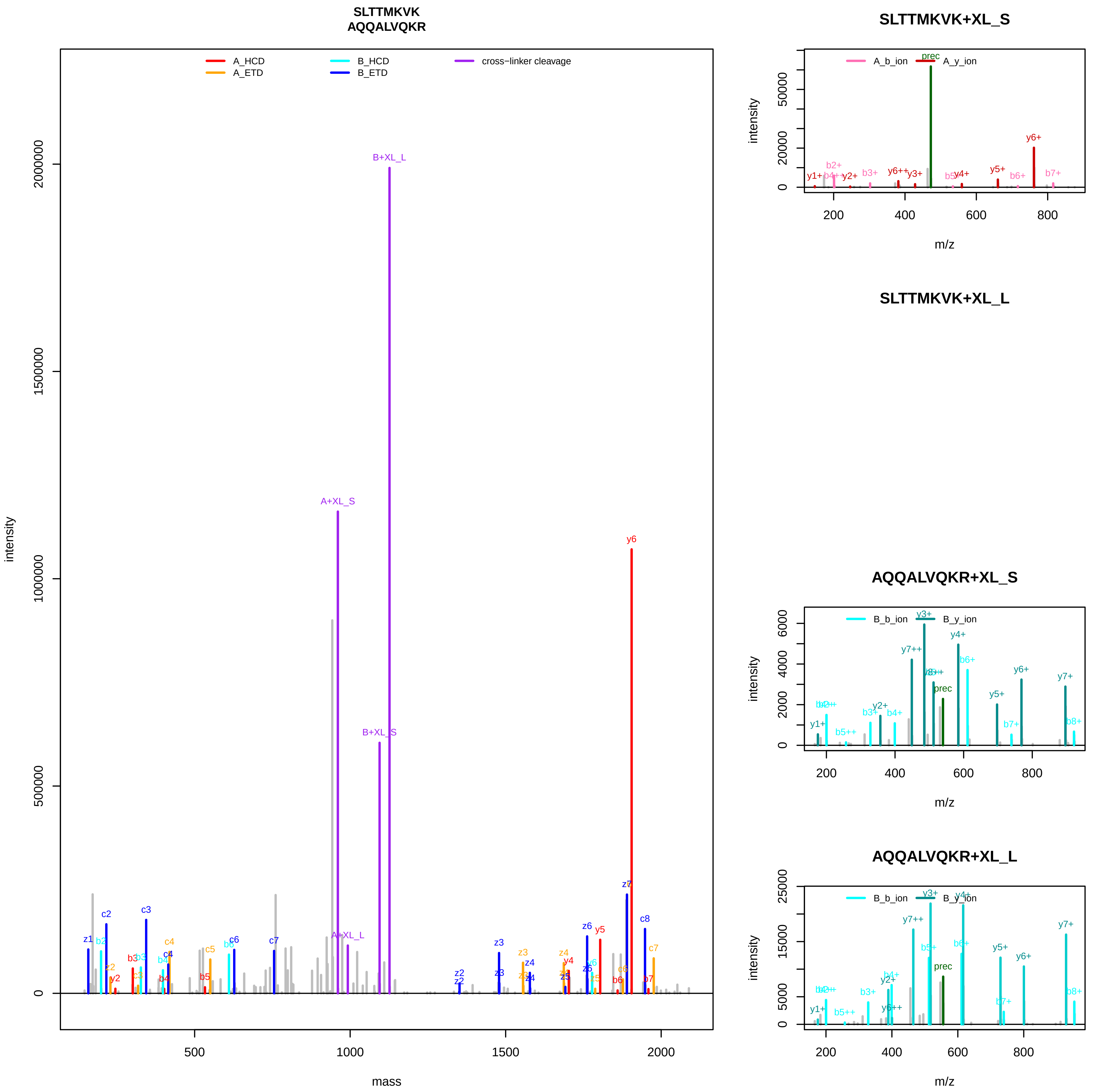
SLTTMKVK
AQQALVQKR
A_HCD
A_ETD
B_HCD
B_ETD
cross−linker cleavage
intensity
mass
0
500000
1000000
1500000
2000000
500
1000
1500
2000
B+XL_L
A+XL_S
B+XL_S
A+XL_L
y6
z7
c8
c7
y5
z6
c6
c7
c3
c2
z1
b2
b3
c4
b4
c5
b5
z3
z4
z2
z3
z4
y4
b6
b7
SLTTMKVK+XL_S
A_b_ion
A_y_ion
prec
intensity
m/z
0
20000
50000
200
400
600
800
y1+
b2+
y2+
b3+
y6++
y3+
b5+
y4+
y5+
y6+
b6+
b7+
SLTTMKVK+XL_L
AQQALVQKR+XL_S
B_b_ion
B_y_ion
y3+
y4+
y7++
b6+
y6+
y7+
y5+
y2+
y1+
b3+
b4+
b5++
b7+
b8+
0
2000
4000
6000
AQQALVQKR+XL_L
0
5000
15000
25000
